# A New Approach for Resolving Conflicts in Actionable Behavioral Rules

**DOI:** 10.1155/2014/530483

**Published:** 2014-08-05

**Authors:** Peng Su, Dan Zhu, Daniel Zeng

**Affiliations:** ^1^School of Mathematics and Computer Science, Dali University, Dali 671003, China; ^2^College of Business, Iowa State University, Ames, IA 50011, USA; ^3^State Key Laboratory of Management and Control for Complex Systems, Institute of Automation, Chinese Academy of Sciences, Beijing 100190, China

## Abstract

Knowledge is considered actionable if users can take direct actions based on such knowledge to their advantage. Among the most important and distinctive actionable knowledge are actionable behavioral rules that can directly and explicitly suggest specific actions to take to influence (restrain or encourage) the behavior in the users' best interest. However, in mining such rules, it often occurs that different rules may suggest the same actions with different expected utilities, which we call conflicting rules. To resolve the conflicts, a previous valid method was proposed. However, inconsistency of the measure for rule evaluating may hinder its performance. To overcome this problem, we develop a new method that utilizes rule ranking procedure as the basis for selecting the rule with the highest utility prediction accuracy. More specifically, we propose an integrative measure, which combines the measures of the support and antecedent length, to evaluate the utility prediction accuracies of conflicting rules. We also introduce a tunable weight parameter to allow the flexibility of integration. We conduct several experiments to test our proposed approach and evaluate the sensitivity of the weight parameter. Empirical results indicate that our approach outperforms those from previous research.

## 1. Introduction

Data mining is the process of discovering patterns in large data sets and then processing these obtained patterns into understandable form for further use [1, 2]. The primary motive behind data mining process is to provide support in decision making process by detecting useful or actionable patterns from a large volume of data [3]. Extensive research has been conducted in data mining to discover hidden patterns from underlying data. However, the knowledge obtained through these data mining techniques is not always useful to the user, and it generally requires significant amount of expertise in postprocessing.

Tradition data mining studies concentrated primarily on predictive mining, where the cause and effect scenario is being described. But this information alone is not sufficient as it does not provide much benefit to the user. What becomes even more interesting and critical to business or organizations is to make the mined patterns or knowledge actionable [4]. Knowledge is considered actionable if users can take direct actions based on such knowledge to their advantage. Actionability is a very important criterion measuring the interestingness of mined patterns. Among the most important and distinctive actionable knowledge are actionable behavioral rules that can directly and explicitly suggest specific actions to take to influence (restrain or encourage) the behavior in the users' best interest [5]. Mining such rules is also an active research area of social computing [6].

In mining such rules, all possible action combinations are considered in turn as a rule's condition. Therefore, candidate actionable behavioral rules may share actions in their conditions. This may lead to several conflicting rules with the same actions, yet different consequences and expected utilities. A rule's expected utility is the utility prediction when the actions suggested by the rule are actually taken. Hence, conflicting rules give different utility predictions for the same actions. Naturally, the rule with the most accurate utility prediction should be reserved for the user and the others should be pruned. Therefore, the key problem is how to accurately evaluate the utility prediction accuracies of conflicting rules.

To resolve conflicting rules, a pruning method was introduced in [5]. Firstly, each general rule whose antecedent is part of another rule is pruned. Subsequently, the remaining rules sharing the same actions are consolidated into a new rule according to their supports. A new rule's expected utility is in fact the weighted average of the remaining rules' expected utilities with their supports as the weights. A major disadvantage of this method lies in the fact that the measure for pruning, namely, antecedent length, is not used consistently. That is to say, antecedent length is only used for some of the conflicting rules.

In this paper, to overcome the shortcomings of the previous method proposed in [5], a new rule ranking method is developed to resolve the conflicts in actionable behavioral rules. More specifically, an integrative measure, which linearly combines the measures of the support and antecedent length, is proposed to evaluate the utility prediction accuracies of conflicting rules. Furthermore, a tunable weight parameter will be introduced in the proposed measure to allow the flexibility of integration. We conduct an experiment to validate our proposed method and evaluate the performance of the proposed method against the previous method and random selection. The experimental results strongly suggest the validity and the superiority of our approach.

Related research on rule pruning in associative classification (AC) inspired our work. Most associative classification algorithms favor rules with large support and confidence values. Another important measure used to determine the precedence of the rules is rule antecedent length. Some AC algorithms, such as those in [7, 8], tend to prefer general rules (those with shorter antecedents) and consequently suffer from poor classification accuracy. On the contrary, other algorithms, such as those in [9, 10], tend to prefer specific rules, reducing the chance of misclassification. However, these methods may lead to more specific rules (i.e., those with longer antecedents). Thabtah argued that ranking of rules should not be limited to support and confidence parameters and proposed a rule ranking procedure that takes into account the class distribution frequency of each rule after considering confidence, support, and rule antecedent length [11].

Mining big data for complex decision making has been a major challenge for next generation data mining. The resolution of the conflicting rule may have significant implications. Actionable knowledge discovery (AKD) not only provides an important tool to decision makers to take appropriate actions but also delivers reliable and actionable outcomes to homeland security and to businesses, to name a few. The rest of the paper is organized as follows.  Section 2 introduces the formal definition of actionable behavioral rules mining.  Section 3 uses an example to illustrate the problem.  Section 4 describes our proposed method.  Section 5 presents our experimental study demonstrating the validity and the superiority of the proposed method.  Section 6 reviews some of the related works. Finally, Section 7 summarizes our contributions and the directions for future research.

## 2. Problem Definitions and Elaborations

To understand the domain in actionable behavioral rule mining, we first introduce some formal definitions listed in Table 1.

Within a behavioral information system, *A*_be_ is a set of behavior attributes describing the behaviors of the entity, and *A*_en_ is a set of environment attributes characterizing the environment in which the entity situates and having some causal influence upon behavior attributes. An action set *S*, also called a |*S*|-action set, is defined as a finite nonempty set of actions such that *t*_1_ · *a* ≠ *t*_2_ · *a* for any *t*_1_, *t*_2_ ∈ *S*. We say that action set *S* holds, if every *t* ∈ *S* holds. We say that observation *o* supports *S*, if *ρ*(*o*, *t* · *a*) = *t* · *v*_*t*_ for every *t* ∈ *S*. The support of *S* is defined as

(1) $$\begin{array}{c} \sup\left(S\right)=\left|\left\{o\in O\;|\;o\;\text{supports}\;S\right\}\right|. \end{array}$$

We call *S* a frequent action set, or frequent |*S*|-action set, with regard to a user-specified threshold referred to as minsup, if sup⁡(*S*)≥ minsup.

In effect, if the value of *a* changes from *v*_*f*_ to *v*_*t*_, *e* = (*a*, *v*_*f*_, *v*_*t*_) takes place. The observation *o* supports (*S*, *e*), where *S* is an action set and *e* is an effect, if *o* supports *S* and *ρ*(*o*, *e* · *a*) = *e* · *v*_*t*_. An effect-probability is defined as a pair *ep* = (*e*, *p*), where *e* is an effect and *p* ∈ [0,1]. We say that an effect-probability *ep* = (*e*, *p*) takes place, if *e* takes place with probability *p*.

We call an actionable behavioral rule *r* an interesting actionable behavioral rule with regard to a user-specified threshold minutil, if util (*r*)≥ minutil.

Please note in the definition, except for the value of o*, that the rest of the observation pertaining to environment or behavior attributes comes from a time period of a certain interval. Based on the recent observation, the forthcoming projection is predicted. Based on the value of o*, the user decides whether or not to take a particular action and how it is going to influence the entity's behavior. Consider a medical scenario where a patient is suffering from a rare disease and the right way to treat this disease is not currently available to the doctors. It is very likely that in a period of time (e.g., a year) the condition of the patient is going to remain unchanged or worsen if proper medication and treatment are not provided. This signifies that a proper action should be taken within a specified time to get a better output. The objective of actionable behavior rule mining is to identify the useful actions that may be applied to improve the projected next observation o*. In the above example the doctors would like to know the actions that can be taken to improve the patients' health condition.

In this paper, we assume that all the attributes (both environmental and behavioral) are categorical, while numerical attributes have been discretized in advance. The behavior attribute values are not restricted to be binary indicating whether a certain behavior occurs or not. The values can also describe how frequent the behavior occurs, the extent of the behavior, and so on. In the next section, we use an example to illustrate some of these concepts and the motivations. For detailed information, please refer to [5] for more explanations and examples.

## 3. An Illustrative Example

Consider an example of a Palestinian terrorist organization Hamas, an Arabic acronym for Harakat al-Muqawma al-Islamiyya. It has carried out sophisticated attacks on Israeli targets and has caused serious troubles for the Israeli government. The Israeli government wants to take some action that will either stop or improve the current situation. For instance, the following prediction rule has been proposed to the government against Hamas:

(2) ({(e1,1,2),(e2,1,3)},{((b1,3,2),19),((b1,3,1),39),((b1,3,3),59)},0.84).

The rule says “if the Israeli government changes the degree of using lethal violence against Hamas from level 1 (not using lethal violence) to level 2 (using periodic lethal violence) and the degree of being in agreement with Hamas from level 1 (negotiation) to level 3 (major concession), the degree of terrorist attacks aiming at domestic targets launched by Hamas will change from level 3 to 2 with 1/9 confidence or 1 with 3/9 confidence or remain unchanged with 5/9 confidence, and the Israeli government will get a utility of 0.84.”

Consider a hypothetical behavioral information system for Hamas organization *I* = (*O*, o*, *A*, *D*, *ρ*), where *O* = {o*, *o*_1_, *o*_2_,…, *o*_10_}, *A*_en_ = {*e*_1_, *e*_2_}, *A*_be_ = {*b*_1_, *b*_2_}, *D*_*e*_1__ = *D*_*e*_2__ = {0,1}, *D*_*b*_1__ = *D*_*b*_2__ = {0,1, 2}, and *ρ* is presented in Table 2 (e.g., *ρ*(*o*_1_, *e*_1_) = 1, *ρ*(*o*_3_, *b*_2_) = 2). The values and the corresponding meanings of the attributes are shown in Table 3.

The problem of mining actionable behavioral rules is to mine all reliable and interesting actionable behavioral rules from a behavioral information system. The threshold minsup is used to assure that the rules are not found by chance. The threshold minutil is used to assure that the rules are sufficiently beneficiary to warrant deliberation by the user.

These actionable rules are important to the user in making decisions. However, rules identified sometimes tend to be conflicting with each other. For example, the following four rules are conflicting, that is,

*r*_1_: ({(*e*_1_, 1,0)}, {((*b*_1_, 2,0), 1/2), ((*b*_1_, 2,1), 1/2), ((*b*_1_, 2, 2), 0)}, 0.10),
*r*_2_: * *({(*e*_1_, 1,0), (*e*_2_, 1,1)}, {((*b*_1_, 2,0), 0), ((*b*_1_, 2,1), 1/3), ((*b*_1_, 2,2), 2/3)}, 0.13),
*r*_3_: * *({(*e*_1_, 1,0), (*e*_2_, 1,1), (*e*_3_, 0,0)}, {((*b*_1_, 2,0), 2/3), ((*b*_1_, 2,1), 1/3), ((*b*_1_, 2,2), 0)}, 0.21),
*r*_4_: * *({(*e*_1_, 1,0), (*e*_4_, 0,0)}, {((*b*_1_, 2,0), 1/3), ((*b*_1_, 2,1), 1/3), ((*b*_1_, 2,2), 1/3)}, 0.17).

These rules are conflicting as they suggest the same standard-action (*e*_1_, 1,0) with different expected utilities, 0.10, 0.13, 0.21, and 0.17. This means that if the user takes the action (*e*_1_, 1,0), we cannot provide him/her with a definite utility estimation. Note that (*e*_2_, 1,1), (*e*_3_, 0,0), and (*e*_4_, 0,0) are nonstandard actions and they cost the government nothing.

Earlier research has dealt with the issues involving conflicting rules. For example, following with the previous example, *r*_1_ and *r*_2_ were pruned first, as they are general rules with regard to *r*_3_. Subsequently, *r*_3_ and *r*_4_ were consolidated into the new rule ({(*e*_1_, 1,0)}, {((*b*_1_, 2,0), 1/2), ((*b*_1_, 2,1), 1/3), ((*b*_1_, 2,2), 1/6)}, 0.19) whose expected utility is the weighted average of the expected utilities of *r*_1_ and *r*_2_ according to their supports. *r*_3_ or *r*_4_ is not pruned as neither is a general rule with regard to the other one.

Previous method prefers the rule with longer rule antecedent length while pruning the general rules. However, the same measure is not used for the rules which do not have general-specific relationship. The nonconsistent use of antecedent length may hurt the performance of the previous method. In addition, there may be another rule selection measure than rule antecedent length, that is, rule support.

## 4. Proposed Approach

The approach we propose to resolve conflicting rules is ranking rules according to their utility prediction accuracies. The highest-order rule should be reserved for the user and the others should be pruned. The key strategy is to find appropriate measure for evaluating conflicting rules.

### 4.1. Candidate Measures for Evaluating Conflicting Rules

There are two main measures in terms of which we can evaluate the utility prediction accuracies of conflicting rules: support and rule antecedent length (i.e., the number of actions in its antecedent). When all other conditions are the same, the higher is a rule's support, the more accurate is its utility prediction. This is due to the fact that higher support can reduce a rule's contingency. When all other conditions are the same, the longer is a rule's antecedent, the more accurate is its utility prediction. The main reason for giving a rule with longer antecedent length more accurate utility prediction than a rule with shorter antecedent length is that the former provides more evidence to make predictions.

However, it is not uncommon that these two measures are conflicting or mutually exclusive. That is to say, it is very likely that a rule may have a longer or shorter antecedent length and lower or higher support than another rule at the same time. Moreover, the precedence of these two parameters is unknown. Finally, the precedence of these two measures will be different for different datasets.

### 4.2. The Rule Ranking Strategy

Due to the conflict between the two measures, we linearly combine them using a tunable weight parameter. In particular, we proposed an integrative measure, score, which combines support and antecedent length to evaluate the utility prediction accuracies of the conflicting rules. This is presented as follows:

(3) $$\begin{array}{c} \text{score}=\alpha \cdot \frac{\sup\left(r\cdot S\right)}{\left(\left|O\right|-1\right)}+\left(1-\alpha \right)\cdot \frac{\left|r\cdot S\right|}{\left|D\right|},\;0\leq \alpha \leq 1, \end{array}$$

where  *r* · *S*: antecedents of rule *r*, *O*: set of behavioral observations, *D*: set of conditional attributes, and *α*: a weight parameter tunable from 0 to 1.

When *α* = 0, the measure depends ultimately on rule antecedent length. When *α* = 1, the measure depends ultimately on support. When 0 < *α* < 1, the measure depends on the combination of these two parameters. The larger *α* is, the more support contributes to the measure. The smaller *α* is, the more rule antecedent length contributes to the measure.

When several rules have the identical highest score, we can choose one of the rules randomly, which possibly in some cases may degrade performance. When the possibility of two or more rules with the same score is relatively high, there should be other parameters to consider in favoring one rule over another in order to limit rule random selection. Thus, we propose a rule ranking procedure that takes into account the class distribution frequency of each conflicting rule after considering score.

This rule ranking procedure adds upon previous rule ranking approaches by looking at the class distribution frequencies in the behavioral information systems and prefers rules whose effects are associated with labels that occur more frequently in the behavioral information systems. The computing procedure of this measure (general) is shown in Algorithm 1.

## 5. Experiments and Results

In this section, we empirically validate the proposed method for resolving conflicting rules. We aim to answer the following five questions. (1) Is the method valid? (2) For which values of *α* does the proposed method outperform the previous method? (3) Does the score-distribution method outperform the score method? (4) For which values of *α* does the proposed method outperform random selection? (5) When *α* increases, how will the performance change?

### 5.1. Experimental Design

To test the validity of our method, we conduct an experiment with the benchmark MAROB datasets (The Minorities at Risk Organizational Behavior Datasets: http://www.cidcm.umd.edu/mar/). The MAROB datasets cover several ethnopolitical organizations in the Middle East and North Africa. The datasets keep track of several attributes on a yearly basis from 1980 to 2004. We extracted three subdatasets about the Hezbollah Organization in Lebanon, the Kurdistan Democratic Party of Iran, and the Iraqi Communist Party, respectively. Utility values of the possible actions and effects from the viewpoints of the corresponding governments were elicited from human analysts and normalized into the range of [−1,1].

Parameter *α* is set to each value in {0,0.1,0.2,…, 1} which is believed to be a good representative of the set of permissible values of *α* ([0,1]).

### 5.2. Evaluation Criterion

We use the mean absolute error (MAE), a standard measure to assess the closeness between predictions and eventual outcomes, as the criterion to evaluate the performance of different methods. The MAE is given by

(4) $$\begin{array}{c} \frac{1}{n}\overset{n}{\underset{i=1}{\sum }}\left|{\text{eu}}_{i}-{\text{au}}_{i}\right|, \end{array}$$

where eu_*i*_ is the expected utility our method or another method estimates and au_*i*_ is the actual utility. Typically, the MAE threshold is set by the domain experts in security informatics to a reasonable value around 0.07 for field evaluation.

### 5.3. Experimental Results

Tables 4 and 5 show the experimental results on the three sub-MAROB datasets with 25 actual action sets that are antecedent of some candidate actionable behavioral rules. Note that there is no other actual action set that is antecedent of any candidate rule. The table entries present the actual utilities, the absolute errors of the proposed score method (Table 4), the proposed score-distribution method (Table 5), the previous method, and random selection with minsup set to 7. For the absolute errors, the means are presented.

Note that although the utilities of actionable behavioral rules-induced actions and effects were assigned by the experts in a subjective fashion, what is actually compared in our experiment is the absolute difference between the actual utility and the expected utility of the action set induced by a rule. For the same action set, different methods will yield different estimated probability distributions over the effects or outcomes. Generally speaking, the closer the estimated distribution of a rule's effects is to the actual realization, the closer the estimated utility is to the actual utility.

From Tables 4 and 5, we can see that the MAE of our approach is much smaller than the validity threshold 0.07. This shows the validity of the proposed method. Furthermore, the proposed method outperforms the previous approach and random selection, when *α* is set to 0 or 0.1. In particular, the proposed method reaches the best performance when *α* is set to 0.1. The score-distribution method outperforms the score method when *α* is set to 0. The two methods yield the same results when *α* is set to the other values, as for these *α* values only one rule has the identical highest score. In general, MAE increases monotonically when *α* becomes larger. This suggests that, in the proposed integrative measure, rule antecedent length has much effect than support. The main reason may be that the small number of instances (fewer than 30) in each dataset leads to the small supports of the rules, and hence the differences among different supports are trivial.

## 6. Related Work

Previous research has looked into actionable knowledge discovery. For example, in discovering actionable knowledge for customer relationship management (CRM), Liu et al. proposed an approach to suggest actions to reclassify a customer from an undesired status to a desired one while postprocessing decision trees to maximum expected net profit [12]. However, these methods could miss some actions with higher net profit. To handle this problem, multiple trees with different subsets of “hard” attributes need to be built. To get optimal actions, the number of trees could be very large, especially when there are many “hard” attributes.

In order to increase profit of an institute to help devise a direct-marketing plan, Yang et al. proposed a lazy approach to use “role models” to generate advice and plans [13]. However, this method failed to provide rules in advance and will incur high computational cost when generating action recommendations. To improve the profitability of a bank, Schrodt proposed action rules constructed from certain pairs of classification rules [14].

Ras and Tzacheva defined interesting action rules as the rules of the lowest cost [15]. Raś et al. proposed a heuristic strategy to construct interesting action rules [16]. Wang et al. combined the action forest algorithm to extract action rules and proposed a heuristic strategy to generate interesting action rules [8]. Tzacheva and Tsay introduced the notion of cost and feasibility of an action rule and proposed a graph based method to search and construct feasible action rules at the lowest cost [17].

Previous studies on mining action rules all produced actionable rules based on a certain pair of classification rules or a single classification rule. A main drawback of these approaches is that some interesting actionable rules can be missed. To address this problem, He et al. proposed another strategy in a support-confidence-cost framework to discover action rules directly from a database [18]. Raś et al. proposed an approach to generate association-type action rules [19]. Subsequently, Raś and Dardzińska proposed a bottom-up strategy to discover action rules without using preexisting classification rules [20].

## 7. Conclusions

Actionable behavioral rules have wide applicability. Finding such rules and applying them are of great importance in the field of data mining [21–27].

In this paper, to resolve conflicts in actionable behavioral rules, we propose a new ranking method using an integrative measure for evaluating the utility prediction accuracies of conflicting rules. The new measure combines the support and antecedent length using a tunable weight parameter. The experimental results strongly suggest the validity and the superiority of our approach.

While we have conducted a preliminary experiment using domain datasets, more comprehensive experiments with many large datasets drawn from various domains can be conducted to validate the generalizability of our findings. For different datasets, the proposed method can tune the weight parameter for the best performance.

## Figures and Tables

**Algorithm 1:**
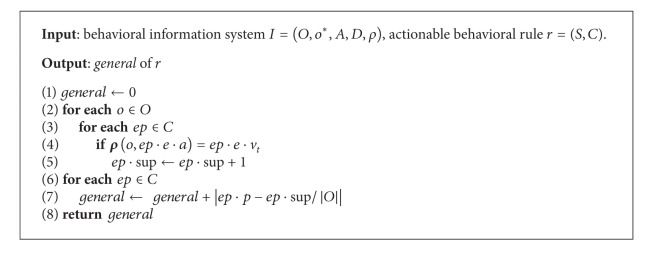


**Table 1:** Concepts and definitions.

Concept	Definition	Explanations
Behavioral information system (BIS)	I = (O, o*, A, D, ρ)	*O*: a finite nonempty set of observations o* ∈ O: the projected next observation *A*: a finite nonempty set of attributes A = A_en_ ∪ A_be_ $D=\underset{a\in A}{⋃}D_{a}$ (D_a_: value domain of attribute *a*) ρ: O × A → *D*: a function associating each observation with a set of attribute values

Action	t = (a, v_f_, v_t_)	a ∈ A_en_ v_f_ = ρ(o*, a) v_t_ ∈ D_a_ If v_f_ ≠ v_t_, t = standard action; else, t = nonstandard action. If the value of a is changed from v_f_ to v_t_, t = (a, v_f_, v_t_) holds.

Effect	e = (a, v_f_, v_t_)	a ∈ A_be_ v_f_ = ρ(o*, a) v_t_ ∈ D_a_

Actionable behavioral rule (ABR)	r = (S, C)	S: an action set C: a finite nonempty set of effect-probabilities $\left|C\right|=\underset{a\in A_{\text{be}}}{\sum }\left|D_{a}\right|$ r = (S, C): if S holds, each effect-probability in C will take place

Expected utility (EU)	util($r)=\underset{t\in S}{\sum }\text{util}(t)+\underset{ep\in C}{\sum }\text{util}\left(ep\cdot e\right)\cdot ep\cdot p$	util(t): utilities of action t util(ep · e): utilities of effect ep · e

**Table 2:** Function *ρ* in behavioral information system.

	*e*_1_	*e*_2_	*b*_1_	*b*_2_
o*	1	1	2	2
*o*_1_	1	1	2	2
*o*_2_	1	0	2	1
*o*_3_	1	0	1	2
*o*_4_	1	0	1	2
*o*_5_	0	1	1	1
*o*_6_	0	1	2	1
*o*_7_	0	1	2	1
*o*_8_	0	0	0	0
*o*_9_	0	0	0	1
*o*_10_	0	0	1	0

**Table 3:** Attributes and meanings.

Code	Meaning	Values	Label
e_1_	Does the state use violence against the organization?	1	Not using lethal violence
		2	Using periodic lethal violence
		3	Using consistent lethal violence against the organization

e_2_	To what degree has the organization succeeded in obtaining government agreement?	1	Negotiation
		2	Some concession
		3	Major concession

b_1_	To what degree does the organization use violence domestically as a strategy?	1	Not using violence as a strategy
		2	Using violence as occasional strategy
		3	Using violence regularly as a strategy

b_2_	To what degree does the organization use violence to target transnational entities as a strategy?	1	Not using violence as a strategy
		2	Using violence as occasional strategy
		3	Using violence regularly as a strategy

**Table 4:** Comparison of the results by score method and other methods.

	Actual utility	Absolute error												
		The previous method	Random selection	The proposed score method										
				*α* = 0	*α* = 0.1	*α* = 0.2	*α* = 0.3	*α* = 0.4	*α* = 0.5	*α* = 0.6	*α* = 0.7	*α* = 0.8	*α* = 0.9	*α* = 1
1	−.01200	0.0500	0.0651	0.0489	0.0400	0.0926	0.0926	0.0926	0.0926	0.0926	0.0880	0.0880	0.0880	0.0880
2	−.01200	0.0471	0.0619	0.0457	0.0400	0.0926	0.0926	0.0926	0.0926	0.0926	0.0880	0.0880	0.0880	0.0880
3	−.00300	0.0933	0.0933	0.0933	0.0933	0.0933	0.0933	0.0933	0.0933	0.0933	0.0933	0.0933	0.0933	0.0933
4	−.00400	0.1244	0.1182	0.1244	0.1244	0.1244	0.1244	0.1244	0.1244	0.1244	0.1120	0.1120	0.1120	0.1120
5	0.0638	0.0000	0.0000	0.0000	0.0000	0.0000	0.0000	0.0000	0.0000	0.0000	0.0000	0.0000	0.0000	0.0000
6	−.00400	0.0500	0.0412	0.0500	0.0500	0.0500	0.0500	0.0500	0.0500	0.0500	0.0356	0.0356	0.0240	0.0240
7	−.01620	0.0000	0.0000	0.0000	0.0000	0.0000	0.0000	0.0000	0.0000	0.0000	0.0000	0.0000	0.0000	0.0000
8	−.006200	0.0533	0.0533	0.0533	0.0533	0.0533	0.0533	0.0533	0.0533	0.0533	0.0533	0.0533	0.0533	0.0533
9	−.00300	0.0000	0.0000	0.0000	0.0000	0.0000	0.0000	0.0000	0.0000	0.0000	0.0000	0.0000	0.0000	0.0000
10	0.1538	0.1467	0.1467	0.1467	0.1467	0.1467	0.1467	0.1467	0.1467	0.1467	0.1467	0.1467	0.1467	0.1467
11	−.01720	0.0267	0.0386	0.0267	0.0267	0.0267	0.0267	0.0267	0.0267	0.0400	0.0533	0.0533	0.0640	0.0640
12	−.01620	0.0933	0.0933	0.0933	0.0933	0.0933	0.0933	0.0933	0.0933	0.0933	0.0933	0.0933	0.0933	0.0933
13	−.00400	0.0267	0.0386	0.0267	0.0267	0.0267	0.0267	0.0267	0.0267	0.0400	0.0533	0.0533	0.0640	0.0640
14	−.08560	0.0000	0.0134	0.0000	0.0000	0.0000	0.0275	0.0275	0.0275	0.0275	0.0259	0.0259	0.0289	0.0289
15	−.00560	0.0000	0.0144	0.0000	0.0000	0.0000	0.0314	0.0314	0.0259	0.0259	0.0259	0.0259	0.0289	0.0289
16	0.0768	0.0200	0.0234	0.0200	0.0200	0.0200	0.0200	0.0200	0.0200	0.0200	0.0200	0.0200	0.0300	0.0300
17	−.08440	0.0000	0.0016	0.0000	0.0000	0.0000	0.0000	0.0000	0.0057	0.0057	0.0057	0.0057	0.0057	0.0057
18	0.0768	0.0200	0.0273	0.0200	0.0200	0.0200	0.0200	0.0200	0.0200	0.0200	0.0343	0.0343	0.0300	0.0300
19	0.0712	0.0200	0.0198	0.0200	0.0200	0.0200	0.0200	0.0200	0.0200	0.0200	0.0200	0.0200	0.0171	0.0171
20	−.00440	0.0933	0.0873	0.0933	0.0914	0.0914	0.0914	0.0800	0.0800	0.0800	0.0800	0.0800	0.0800	0.0800
21	−.01000	0.0462	0.0455	0.0462	0.0457	0.0457	0.0457	0.0457	0.0440	0.0440	0.0440	0.0440	0.0440	0.0440
22	0.1168	0.0171	0.0207	0.0171	0.0171	0.0171	0.0171	0.0171	0.0171	0.0171	0.0171	0.0171	0.0300	0.0300
23	−.00600	0.0629	0.0809	0.0629	0.0629	0.0629	0.0629	0.1057	0.1057	0.1057	0.1057	0.0933	0.0933	0.0933
24	−.06660	0.0006	0.0026	0.0006	0.0006	0.0006	0.0006	0.0006	0.0006	0.0006	0.0006	0.0086	0.0086	0.0086
25	0.1940	0.1371	0.1252	0.1371	0.1371	0.1371	0.1371	0.1371	0.1371	0.1057	0.1057	0.0933	0.0933	0.0933
26	−.00760	0.0790	0.0773	0.0790	0.0790	0.0790	0.0790	0.0790	0.0854	0.0854	0.0854	0.0950	0.0950	0.0950

Mean		0.0465	0.0496	0.0464	**0.0457 **	0.0498	0.0520	0.0532	0.0534	0.0532	0.0534	0.0531	0.0543	0.0543

**Table 5:** Comparison of the results by score-distribution method and other methods.

	Actual utility	Absolute error												
		The previous method	Random selection	The proposed score-distribution method										
				*α* = 0	*α* = 0.1	*α* = 0.2	*α* = 0.3	*α* = 0.4	*α* = 0.5	*α* = 0.6	*α* = 0.7	*α* = 0.8	*α* = 0.9	*α* = 1
1	−.01200	0.0500	0.0651	0.0400	0.0400	0.0926	0.0926	0.0926	0.0926	0.0926	0.0880	0.0880	0.0880	0.0880

2	−.01200	0.0471	0.0619	0.0400	0.0400	0.0926	0.0926	0.0926	0.0926	0.0926	0.0880	0.0880	0.0880	0.0880
3	−.00300	0.0933	0.0933	0.0933	0.0933	0.0933	0.0933	0.0933	0.0933	0.0933	0.0933	0.0933	0.0933	0.0933
4	−.00400	0.1244	0.1182	0.1244	0.1244	0.1244	0.1244	0.1244	0.1244	0.1244	0.1120	0.1120	0.1120	0.1120
5	0.0638	0.0000	0.0000	0.0000	0.0000	0.0000	0.0000	0.0000	0.0000	0.0000	0.0000	0.0000	0.0000	0.0000
6	−.00400	0.0500	0.0412	0.0500	0.0500	0.0500	0.0500	0.0500	0.0500	0.0500	0.0356	0.0356	0.0240	0.0240
7	−.01620	0.0000	0.0000	0.0000	0.0000	0.0000	0.0000	0.0000	0.0000	0.0000	0.0000	0.0000	0.0000	0.0000
8	−.006200	0.0533	0.0533	0.0533	0.0533	0.0533	0.0533	0.0533	0.0533	0.0533	0.0533	0.0533	0.0533	0.0533
9	−.00300	0.0000	0.0000	0.0000	0.0000	0.0000	0.0000	0.0000	0.0000	0.0000	0.0000	0.0000	0.0000	0.0000
10	0.1538	0.1467	0.1467	0.1467	0.1467	0.1467	0.1467	0.1467	0.1467	0.1467	0.1467	0.1467	0.1467	0.1467
11	−.01720	0.0267	0.0386	0.0267	0.0267	0.0267	0.0267	0.0267	0.0267	0.0400	0.0533	0.0533	0.0640	0.0640
12	−.01620	0.0933	0.0933	0.0933	0.0933	0.0933	0.0933	0.0933	0.0933	0.0933	0.0933	0.0933	0.0933	0.0933
13	−.00400	0.0267	0.0386	0.0267	0.0267	0.0267	0.0267	0.0267	0.0267	0.0400	0.0533	0.0533	0.0640	0.0640
14	−.08560	0.0000	0.0134	0.0000	0.0000	0.0000	0.0275	0.0275	0.0275	0.0275	0.0259	0.0259	0.0289	0.0289
15	−.00560	0.0000	0.0144	0.0000	0.0000	0.0000	0.0314	0.0314	0.0259	0.0259	0.0259	0.0259	0.0289	0.0289
16	0.0768	0.0200	0.0234	0.0200	0.0200	0.0200	0.0200	0.0200	0.0200	0.0200	0.0200	0.0200	0.0300	0.0300
17	−.08440	0.0000	0.0016	0.0000	0.0000	0.0000	0.0000	0.0000	0.0057	0.0057	0.0057	0.0057	0.0057	0.0057
18	0.0768	0.0200	0.0273	0.0200	0.0200	0.0200	0.0200	0.0200	0.0200	0.0200	0.0343	0.0343	0.0300	0.0300
19	0.0712	0.0200	0.0198	0.0200	0.0200	0.0200	0.0200	0.0200	0.0200	0.0200	0.0200	0.0200	0.0171	0.0171
20	−.00440	0.0933	0.0873	0.0933	0.0914	0.0914	0.0914	0.0800	0.0800	0.0800	0.0800	0.0800	0.0800	0.0800
21	−.01000	0.0462	0.0455	0.0467	0.0457	0.0457	0.0457	0.0457	0.0440	0.0440	0.0440	0.0440	0.0440	0.0440
22	0.1168	0.0171	0.0207	0.0171	0.0171	0.0171	0.0171	0.0171	0.0171	0.0171	0.0171	0.0171	0.0300	0.0300
23	−.00600	0.0629	0.0809	0.0629	0.0629	0.0629	0.0629	0.1057	0.1057	0.1057	0.1057	0.0933	0.0933	0.0933
24	−.06660	0.0006	0.0026	0.0006	0.0006	0.0006	0.0006	0.0006	0.0006	0.0006	0.0006	0.0086	0.0086	0.0086
25	0.1940	0.1371	0.1252	0.1371	0.1371	0.1371	0.1371	0.1371	0.1371	0.1057	0.1057	0.0933	0.0933	0.0933
26	−.00760	0.0790	0.0773	0.0790	0.0790	0.0790	0.0790	0.0790	0.0854	0.0854	0.0854	0.0950	0.0950	0.0950

Mean		0.0465	0.0496	0.0458	**0.0457**	0.0498	0.0520	0.0532	0.0534	0.0532	0.0534	0.0531	0.0543	0.0543
